# Public Baths in the United States

**Published:** 1905-02-04

**Authors:** 


### PUBLIC BATHS IN THE UNITED STATES.

Europe showed the way to America in the provision of public baths for the people, but since the example has been set it has been ardently followed. In every city of importance, from New York to San Francisco, including many active towns whose names are almost unknown to British ears, baths are provided free, or at less than the cost of maintenance, by the State or the town, or by some association interested in the welfare of the people. To enumerate all these would be uninteresting, but we may quote the characteristics of a few to show the energy with which Americans have taken up the idea.

Seaboard and lakeside cities have expended much money in the fitting up of bathing beaches, with separate sections and dressing houses for men and boys, women and girls. In the summer the visitors to these beaches can everywhere be reckoned by thousands, and the faithful few who bathe all the year round in the open can be found in Boston and Chicago, as they can on the banks of the Serpentine. Besides these beaches, open floating bathing-tanks are frequent, modelled rather on the fashions of Continental watering-places than on anything English. The floors of these are mounted on empty barrels, and rise and fall with the tide, while the sides are made of open slat-work, permitting the automatic renewing of the water twice a day as the tide comes in. These bathing tanks are a great convenience for those who wish to learn to swim. It is rather strange that in Britain, where so many people go annually to the sea-side, such conveniences for the timid and the untaught should be so rare.

We are more in touch with the indoor bathing facilities of the United States, though even here there are some novelties. One thing that strikes a Briton in the American baths is the preference shown there for the shower rather than the tub. With us a bath means almost invariably the immersing of the person, and shower-baths are looked upon as a tonic rather than as an aid to cleanliness. But as an observer of the American system has remarked, it is really easier to cleanse oneself by means of a shower or spray-bath than by any other, and in public baths this method is both more speedy and more convenient. If the bather wets and soaps himself all over, then turns on the shower, which is regulated so that he does not receive either very cold or very warm water, he will be thoroughly cleansed in a very few minutes, and there will be no tub to be swilled out by the bath-attendant before another bather can go in. Thus both time and labour are saved. A wise regulation, which is almost universal is that anyone who wishes to enter the swimming-bath of these establishments, must take a cleansing bath first, and the time allowed for the latter is three minutes for men and boys and five for women and girls.

It may be objected that for the female sex the shower-bath has the disadvantage of wetting the hair. But this trouble is got over in two ways. In many baths the shower really means the ring bath, in which the water flows through a perforated ring or yoke, which is placed on the shoulders. Thus the body is cleansed without the head being wet. But in Brooklyn, the bath authorities are so obliging as to provide steam-heated hair-drying rooms for their lady patrons. This is only one example of the thought that is given to the arrangement of these public baths. Of this perhaps the most remarkable example is to be found in the Sutro baths, in San Francisco, built by Mr. Adolf Sutro, a prominent citizen of the town. These are sea-water baths, consisting of six tanks, of which one is kept at the natural temperature of the water, while the others are heated in varying degrees. The length of the baths is within six inches of 500 feet, and the width rather over 250 feet. The baths will hold 25,000 bathers and 7,000 spectators. The number of private dressing-rooms is 517, while the nine club-rooms accommodate more than 1,000 more. Toboggan slides, spring boards, trapezes, and swinging rings are provided in connection with the tanks. Besides the salt-water tanks there is a fresh-water plunge tank, and a number of shower baths. A fully-equipped laundry is attached to the baths, with the means of doing up 20,000 suits and 40,000 towels a day. There is also a restaurant connected with it, which can seat 1,000 persons at a time, while the kitchen staff is calculated to meet the wants of 6,000 visitors.

These American baths must be a liberal education to the immigrants from Europe, many of whom have not lived before in environments where they were invited to cleanliness. The kind of people who go to each varies according to the situation. Some are patronised largely by negroes, others by Jews, others again by Italians. One of the Boston baths is patronised almost exclusively by Irish. These are the baths of the working people, and are either entirely free or cost only a few cents for the hire of a towel and bathing suit. In some of the places where Russian and other elaborate baths are obtainable, a higher charge is made, but even there 25 cents—*i.e.*, a little over a shilling—is regarded as a sufficient price.

